# Identification of Deep-Intronic Splice Mutations in a Large Cohort of Patients With Inherited Retinal Diseases

**DOI:** 10.3389/fgene.2021.647400

**Published:** 2021-03-02

**Authors:** Xinye Qian, Jun Wang, Meng Wang, Austin D. Igelman, Kaylie D. Jones, Yumei Li, Keqing Wang, Kerry E. Goetz, David G. Birch, Paul Yang, Mark E. Pennesi, Rui Chen

**Affiliations:** ^1^Verna and Marrs McLean Department of Biochemistry and Molecular Biology, Baylor College of Medicine, Houston, TX, United States; ^2^Human Genome Sequencing Center, Baylor College of Medicine, Houston, TX, United States; ^3^Department of Molecular and Human Genetics, Baylor College of Medicine, Houston, TX, United States; ^4^Department of Ophthalmology, Casey Eye Institute, Oregon Health & Science University, Portland, OR, United States; ^5^Retina Foundation of the Southwest and Department of Ophthalmology, University of Texas Southwestern Medical Center, Dallas, TX, United States; ^6^Office of the Director, National Eye Institute/National Institutes of Health, Bethesda, MD, United States

**Keywords:** inherited retinal dystrophies, whole-genome sequencing, splicing, deep-intronic mutations, minigenes

## Abstract

High throughput sequencing technologies have revolutionized the identification of mutations responsible for a diverse set of Mendelian disorders, including inherited retinal disorders (IRDs). However, the causal mutations remain elusive for a significant proportion of patients. This may be partially due to pathogenic mutations located in non-coding regions, which are largely missed by capture sequencing targeting the coding regions. The advent of whole-genome sequencing (WGS) allows us to systematically detect non-coding variations. However, the interpretation of these variations remains a significant bottleneck. In this study, we investigated the contribution of deep-intronic splice variants to IRDs. WGS was performed for a cohort of 571 IRD patients who lack a confident molecular diagnosis, and potential deep intronic variants that affect proper splicing were identified using SpliceAI. A total of six deleterious deep intronic variants were identified in eight patients. An *in vitro* minigene system was applied to further validate the effect of these variants on the splicing pattern of the associated genes. The prediction scores assigned to splice-site disruption positively correlated with the impact of mutations on splicing, as those with lower prediction scores demonstrated partial splicing. Through this study, we estimated the contribution of deep-intronic splice mutations to unassigned IRD patients and leveraged *in silico* and *in vitro* methods to establish a framework for prioritizing deep intronic variant candidates for mechanistic and functional analyses.

## Introduction

Inherited retinal diseases (IRDs) are a diverse set of Mendelian disorders that are clinically heterogeneous and are a major cause of inherited blindness. They are caused by the progressive deterioration or the early loss of cells fundamental for the normal function of the retina (den Hollander et al., 2010). Decades of studies have illustrated the genetic basis of IRDs by revealing mutations in over 200 genes (Ellingford et al., 2016). Conventional molecular diagnoses focus on coding and flanking canonical splice site sequences that harbor the majority of genetic defects, enabling a molecular diagnosis discovery rate of 66% for Stargardt disease (Zaneveld et al., 2015), 60% for retinitis pigmentosa (RP; Zhao et al., 2015), 75% for Leber congenital amaurosis (LCA; Wang et al., 2015), and 70% for Usher syndrome (Jiang et al., 2015). Despite that the applications of next-generation sequencing (NGS) for molecular diagnostics increasing the diagnostic yield for individuals with IRDs to 70% (Ellingford et al., 2016), 30% of patients with a clinical diagnosis of IRD still have no causal mutation identified. The remaining unsolved cases could be attributed to various reasons such as (1) variants in known retinal disease genes that are missed by sequencing or not interpreted as pathogenic, (2) variants in genes have not yet been associated with retinal phenotypes, or (3) novel genetic mechanisms that have not been linked to Mendelian disease. In this study, we evaluated the contribution of deep-intronic splicing variants in known retinal disease genes in unsolved IRD cases.

Precise pre-mRNA splicing is essential for appropriate protein translation, and defective splicing has been increasingly recognized as disease-causing (Krawczak et al., 2007; Padgett, 2012; Singh and Cooper, 2012; Pedrotti and Cooper, 2014; Sterne-Weiler and Sanford, 2014; Chabot and Shkreta, 2016; Scotti and Swanson, 2016). Predicting the impact of splicing-altering variants is essential to understanding human diseases, as mutations that affect pre-mRNA splicing contribute to at least 15% of disease-causing mutations (Krawczak et al., 1992) and, in some genes, up to 50% of all mutations described in some genes (Teraoka et al., 1999; Ars et al., 2000). Due to the size of intronic regions, identifying deep intronic variants that affect splicing is challenging. The recent applications of whole-genome sequencing (WGS) to clinical screening studies enable the investigation of noncoding variation and identification of pathogenic deep intronic variants that lie >100 bp away from the nearest canonical splice sites in several IRD genes, such as *ABCA4* (Albert et al., 2018; Fadaie et al., 2019; Nassisi et al., 2019; Sangermano et al., 2019) and *RPGRIP1* (Jamshidi et al., 2019).

To identify disease-causing deep intronic mutations and investigate their effects on splicing, we need to combine sequencing of intronic or whole genomic regions with approaches that enable the assessment of a variant’s effect on mRNA transcript. The ideal method to determine how a variant affects splicing is to perform reverse transcription PCR (RT-PCR) on transcripts affected by the variant using extracted RNA from the affected tissue of patients. However, direct RNA analyses on patient biopsy materials are greatly limited, especially in the case of IRDs, where it is not practical to biopsy retinal tissues. One alternative to circumvent these obstacles is to use *in vitro* minigenes, which directly determine whether single nucleotide polymorphisms (SNPs) disrupt splicing regulation. Many previous studies have validated the minigene assay as a viable approach to evaluate splicing alterations (Spickett et al., 2016; Zernant et al., 2018; Bauwens et al., 2019; Toulis et al., 2020).

In recent years, the development of a new computational tool based on the deep-learning network, SpliceAI, has greatly improved our ability to predict cryptic splice variants. SpliceAI is different from previous bioinformatic approaches that focused on shorter nucleotide windows within or close to exon-intron junctions. Instead, it uses a wider window (up to 10 k base pairs) to predict splice junctions (Jaganathan et al., 2019). It outperforms previous tools in sensitivity and specificity (Wai et al., 2020). However, despite a dramatically improved performance, there is significant room for improvement. For example, with a score cutoff ≥0.5, SpliceAI has a sensitivity of 71% when the variant is near exons, but the sensitivity drops to 41% with an unknown specificity when the variant is in the deep intronic regions (Jaganathan et al., 2019). To overcome this challenge, we supplemented SpliceAI predictions with *in vitro* functional assays to identify and validate putative deep-intronic splicing variants.

In this study, we focused on the contribution of deep-intronic variants that disrupt splicing to diseases of 571 patients diagnosed with IRDs but had no molecular diagnosis by conventional clinical molecular diagnostic approaches, which provide information restricted to the protein-coding exons and exon-intron boundaries. We leveraged the *in silico* SpliceAI prediction on variant-induced splicing alterations and an *in vitro* minigene system to validate the predictions made on splicing alterations. All patient variants that we identified based on our prioritization strategy demonstrated RNA splicing patterns that deviate from wild-type controls, indicating the deleterious effects of identified variants.

## Materials And Methods

### Clinical Diagnosis and Patient Recruitment

All probands discussed herein were clinically diagnosed with retinal diseases following a thorough ophthalmologic examination by a qualified collaborating ophthalmologist. This study was approved by the institutional ethics boards at each affiliated institution and adhered to the tenets of the declaration of Helsinki. Before blood collection, all probands and family members provided written informed consent for DNA analysis and received genetic counseling in accordance with guidelines. DNA samples from patients and available relatives were obtained using the Qiagen blood genomic DNA extraction kit (Qiagen, Hilden, Germany).

### Variant Annotation and Variant Prioritization for Splicing Functional Validation

All patients in our cohort first underwent panel testing to identify disease-causing mutations. For the ones who lack a confident molecular diagnosis, we assigned them as “unsolved” and proceed to further in-depth analyses of these patients. All patient DNAs which underwent WGS were further studied at the Human Genome Sequencing Center, Baylor College of Medicine. WGS data were processed using a pipeline modified from our previous WES data analysis pipeline (Soens et al., 2016). Briefly, NGS sequencing reads were aligned to the human genome assembly (hg19) with BWA (Li and Durbin, 2009). Single nucleotide variants/small insertion-deletion variants (SNVs/INDELs) were identified using GATK 4, while structure variants/copy number variants (SVs/CNVs) were identified using CNVnator v0.3, Delly v0.7.8, Lumpy 0.2.13, and Manta 1.2.2. A population frequency threshold of 0.5% was used to filter out common variants that occur too frequently to be the cause of rare IRDs. SNVs/INDELs that were mapped to the coding region were annotated with ANNOVAR and searched against the dbNSFP 3.5a database. The conservation of the remaining variants was estimated based on phastCons.hg19.100way downloaded from UCSC Genome Browser (Pollard et al., 2010). The effect of coding variants was predicted using CADD v1.3 (Kircher et al., 2014; Xu et al., 2017; Rentzsch et al., 2019). SVs/CNVs were annotated to the RefSeq gene database and filtered by svtyper 0.7.0 (score cutoff 100; Chiang et al., 2015; Ebler et al., 2017). Raw bam files that contained candidate SVs/CNVs were checked manually through IGV to rule out potential false-positive calls from mapping errors and sequencing errors.

### Variant Prioritization Strategy for Splicing Functional Validation

Our scheme for variant prioritization for splicing functional validation was as follows (Supplementary Figure S1). Starting with single-nucleotide variant (SNVs) captured by WGS from all 571 unsolved IRD patients, we filtered and annotated genomic alterations with a custom pipeline and predicted the effects of intronic variants on splicing using Splice AI (Jaganathan et al., 2019). SpliceAI (spliceai-1.2.1) was run on the variants passing the allele frequency filtering. The *in silico* splicing variant predictor SpliceAI assigns a score to each variant, providing predictions of how the variant affects splicing. The score value lies between the range of 0 and 1, and the higher the score, the more confident we are that the candidate variant may affect splicing. The candidate splicing variants were restricted to those found in previously reported IRD genes and were selected with a SpliceAI prediction score cutoff of 0.5 (Supplementary Table S1). Then variants were analyzed based on genes’ inheritance patterns. If the detected splicing variant is on IRD genes that are associated with dominant diseases or are X-linked hemizygous, only one hit of the splicing variant was considered to be sufficient. The variants on genes that are associated with recessive diseases require one more allele that is either in the coding region or also affects splicing in addition to the detected splicing variant. The candidates were further filtered by their distance from the exon-intron junctions (>10 bp) to eliminate any variants that are too close to the canonical splice sites, leaving only the deep-intronic candidate splicing variants.

### Minigene Molecular Cloning, Transfection, and RT-PCR

Next, to assess the effects on splicing of the prioritized variants, we used an established minigene reporter assay called the RHCglo minigene (Singh and Cooper, 2012). A genomic region from each patient, consisting of the exon closest to the candidate splicing variant (the test exon), the predicted cryptic exon and between approximately 150 base pairs of surrounding introns, was PCR-amplified (Supplementary Figure S2). A wild-type (WT) amplicon and a variant (Var) amplicon were obtained by using heterozygous patient DNA as a template, or a wild-type sequence was amplified from control placenta DNA when the patient was homozygous for a variant. The PCR products obtained were cloned into the RHCglo vector. For the patients who have more than one mutation in the PCR-amplified region based on Sanger sequencing results, site-directed mutagenesis was performed using the wild-type-amplicon-containing vector as the template. The impact of splicing variants was examined by transfecting plasmids into HEK293 cells, followed by an RT-PCR assay as previously described (Soens et al., 2017). The intensities of DNA bands were quantitated using ImageJ Gel Analysis program.

### Sanger Sequencing

Sequencing was performed as previously described (Soens et al., 2016), and primer designs are described in Supplementary Table S1. Sanger sequencing was performed to confirm (1) proper variant segregation within patients’ family, (2) the authenticity of variants identified by NGS, and (3) the sequence of gel-extracted RT-PCR bands.

## Results

To assess the prevalence of deep-intronic splicing mutations in patients with IRD, we analyzed the WGS data from a cohort of 571 IRD patients whose mutations have not been identified by panel testing. As described in the Materials and Methods section, six deleterious deep-intronic variants (Table 1) were identified in eight probands (Table 2). One variant has been previously reported, and five are novel mutations in regions beyond 50 bp of the exon-intron boundary in known IRD genes.

**Table 1:** Patient variant information.

Patient ID	Gene	Chromosomal position	cDNA variant	Zygosity	Variant type	Protein variant	Novel variant?
DGB288	ADGRV1	chr5:89979702 T>TA	c.5965dupA	heterozygous	frameshift	p.V1988fs	known	
		chr5:90099416A>G	c.14661+717A>G	heterozygous	splicing		novel		
DGB289	USH2A	chr1:215963510C>T	c.10073C>T	heterozygous	nonsynonymous	p.C3358Y	known	
		chr1:216041166C>G	c.8682-654C>G	heterozygous	splicing		novel		
MEP337	USH2A	chr1:215901574G>A	c.11864G>A	heterozygous	nonsense	p.W3955X	known	
		chr1:216041166C>G	c.8682-654C>G	heterozygous	splicing		novel		
MEP344	OPA1	chr3:193362516A>G	c.1608+622A>G	heterozygous	splicing		novel
NEI4320	RPGRIP1	chr14:21793128A>C	c.2114A>C	heterozygous	nonsynonymous	p.Q705P	novel	
		chr14:21793624A>G	c.2367+82A>G	heterozygous	splicing		novel		
MEP129	CNGB3	chr8:87656008AG>A	c.1148delC	heterozygous	frameshift	p.T383fs	known	
		chr8:87617644G>A	c.1663-1205G>A	heterozygous	splicing		novel		
MEP130	CNGB3	chr8:87656008AG>A	c.1148delC	heterozygous	frameshift	p.T383fs	known	
		chr8:87617644G>A	c.1663-1205G>A	heterozygous	splicing		novel		
MEP105	PCDH15	chr10:55955474G>T	c.1163G>T	heterozygous	nonsynonymous	p.L429P	novel	
		chr10:55597057 T>G	c.3998+3,023 T>G	heterozygous	splicing		novel		

**Table 2:** Clinical data.

Patient ID	Clinical diagnosis	Sex	Age	Race	Age of onset (y.o.)	BCVA		Other
						Right	Left	
MEP337	Usher II	F	14 y.o.	Caucasian	7	20/25-	20/25-+2	Congenital sensorineural hearing loss
DGB289	RP	F	69 y.o.	Caucasian	5–6	20/125	LP	
MEP344	Optic atrophy	M	16 y.o.	Caucasian	8	20/100-1	20/100	
DGB288	Usher II	F	70 y.o.	Caucasian	59^∗^	20/200	20/32	Congenital sensorineural hearing loss
NEI317	CRD	F	65 y.o.	Caucasian	60			
MEP129	Achromatopsia	M	5 y.o.	Hispanic	2 m.o.	20/125	20/100	
MEP130	Achromatopsia	F	13 y.o.	Hispanic	1 m.o.	20/150	20/150-	
MEP105	CRD	M	67 y.o.	Caucasian	42	20/25+2	20/30-2+2	

LP, light perception (the ability to perceive the difference between light and dark); BCVA, best corrected visual acuity; RP, retinitis pigmentosa; CRD, cone-rod dystrophy.

∗ Born with hearing loss.

### Identification of One Known Deep-Intronic Splicing Variant in Two IRD Patients

**Figure 1:**
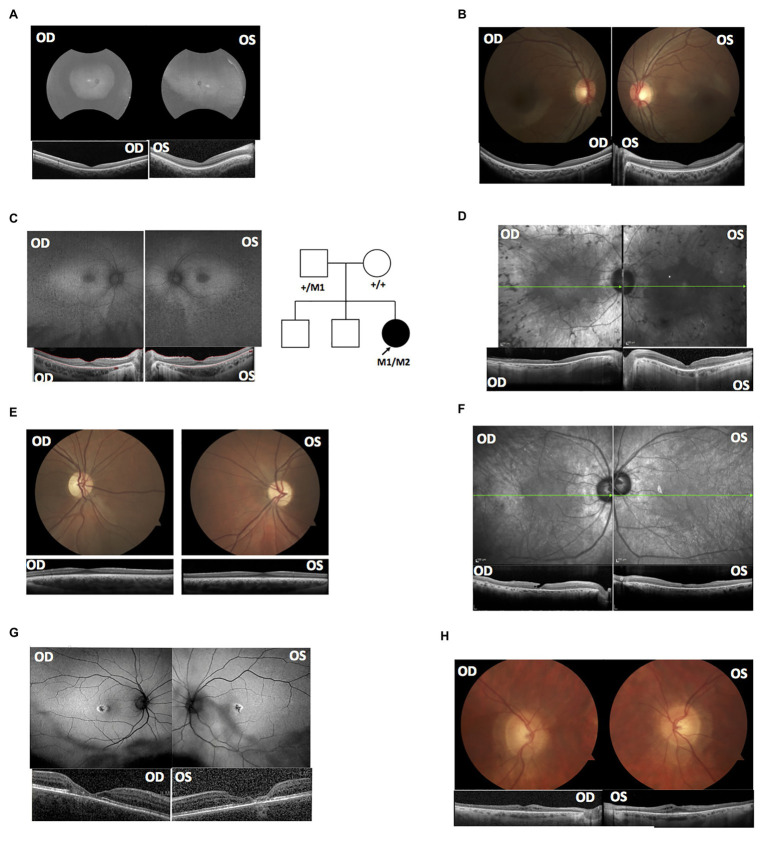
Clinical data supported the splicing variants’ deleterious effects based on known genotype-phenotype associations. **(A)** Fundus autofluorescence (AF) and optical coherence tomography (OCT) of MEP129. **(B)** OCT and fundus images of MEP130. **(C)** AF and OCT of MEP337; the right panel shows the pedigree of proband MEP337. Circles represent females; squares represent males. Empty shapes are unaffected relatives. The arrow indicates proband MEP337. M1: c.G11864A; M2: c.8682-654C>G. **(D)** AF and OCT of DGB289. **(E)** OCT and fundus images of MEP344. **(F)** AF and OCT of DGB288. **(G)** AF and OCT of NEI4320. **(H)** OCT and fundus images of MEP105.

MEP129 and MEP130 are two affected siblings from the same family and were diagnosed with achromatopsia (Figures 1A,B). WGS of patient DNA revealed that they both have two heterozygous variants, c.1148delC and c.1663-1205G>A, in exon 7 and intron 11 of *CNGB3*, respectively. Mutation in *CNGB* is the most common cause of achromatopsia. The coding variant c.1148delC is a recurrent pathogenic mutation found in *CNGB3* (Kohl et al., 2005), while the c.1663-1205G>A intronic splicing variant was previously observed in 18 patients (Weisschuh et al., 2020). Based on the results of *in vitro* splicing assays, the c.1663-1205G>A allele yielded two RT-PCR products; the major product comprised not only exons 14 and 15, but also a pseudo-exon of 34 nucleotides spliced between both canonical exons, while the minor product corresponds to the correctly processed transcript (Weisschuh et al., 2020).

### Identification and Validation of Five Novel Deep-Intronic Splicing Variants

**Figure 2:**
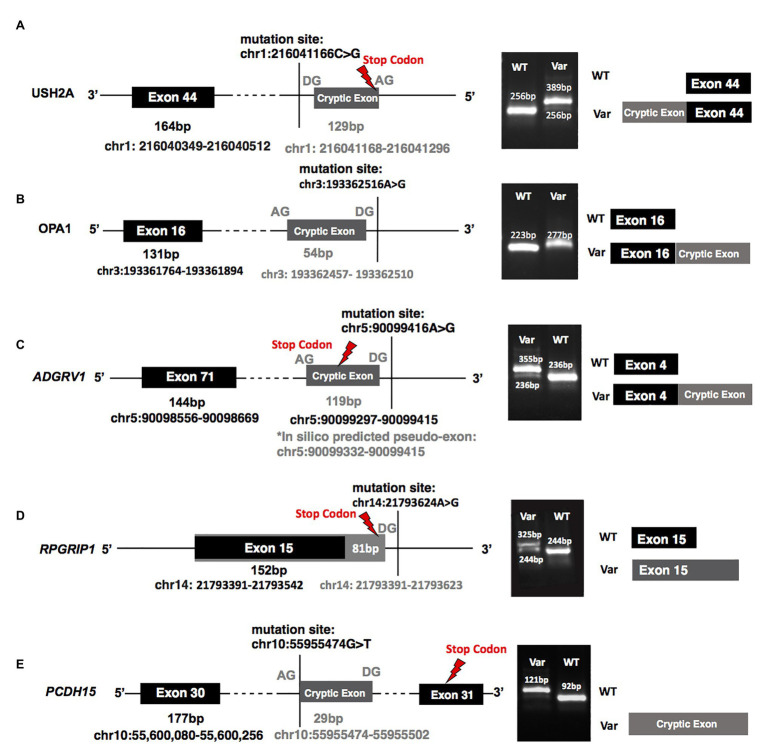
A minigene splicing assay reveals variant-induced aberrant splicing. **(A–E)** Demonstrate the splicing results of variants identified in patients: **(A)** MEP337 and DGB289, **(B)** MEP344, **(C)** DGB288, **(D)** NEI4320, and **(E)** MEP105. The left panels of **(A–E)** plot the chromosomal positions of mutations as well as those of the cryptic exons and the stop codons generated by each variant; the middle panels show gel electrophoresis of reverse transcription PCR (RT-PCR) products of all tested minigenes. All the gel bands were longer than the control exons and predicted exons as RT-PCR primers were designed to include partial regions (92 bp) of the first and the third exons (the exons before and after the replaceable exon as shown in Supplementary Figure S2) in the minigene vector. The diagrams in the right panel, which are not to scale, are provided as a schematic of the variant-induced changes in transcript configuration. WT, splicing results of wild-type control; Var, splicing results of identified variants.

Among the five newly identified deep-intronic splicing variants, four were predicted to create both cryptic donor and acceptor sites, and one was predicted to activate a novel cryptic acceptor site while disrupting the original canonical acceptor site (Table 2). To further confirm the prediction made by *in silico* tools and reveal the functional impact of identified candidate variants on mRNA splicing, we performed a functional splicing assay using the *in vitro* RHCglo minigene system (Singh and Cooper, 2012). Consistent with the *in silico* prediction, RT-PCR products indicated that all predicted candidate splicing variants produced new bands with different lengths compared to the wild-type controls (Figure 2). Detailed information for each mutant allele is described below.

**Figure 3:**
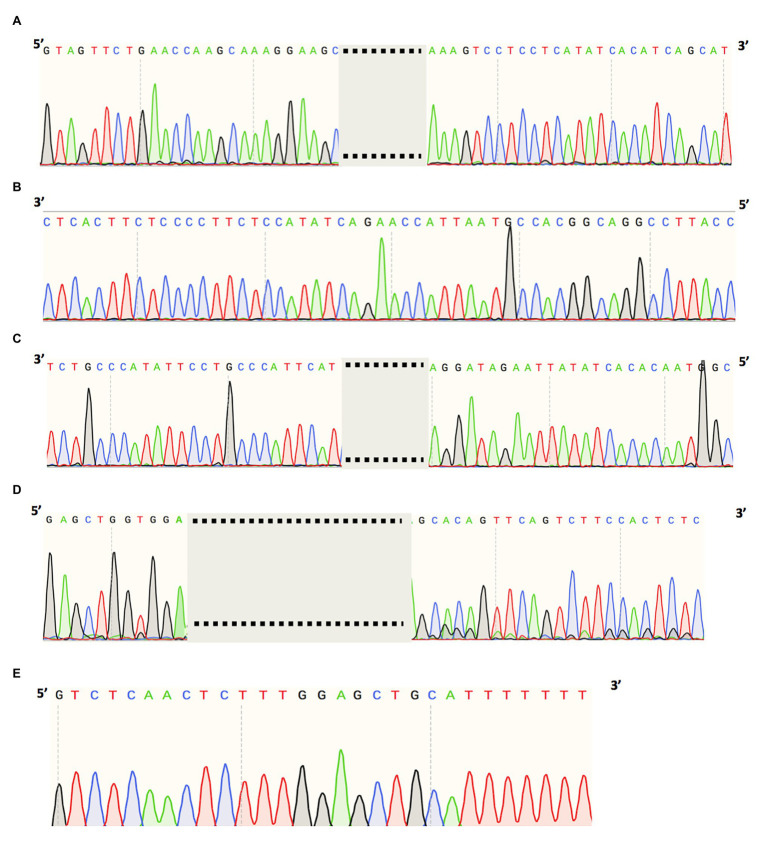
Cryptic exon sequences of **(A)** *USH2A* splice variant (chr1:216041166C>G) of MEP337 and DGB289, **(B)** *OPA1* splice variant (chr3:193362516A>G) of MEP344, **(C)** *ADGRV1* splice variant (chr5:90099416A>G) of DGB288, **(D)** *RPGRIP1* splice variant (chr14:21793624A>G) of NEI4320, and **(E)** *PCDH15* splice variant (chr10:55597057T>G) of MEP105.

A heterozygous deep-intronic splicing variant (c.8682-654C>G) was found in intron 43 of *USH2A* in two unrelated patients diagnosed with Usher syndrome type II (Usher II), MEP337 and DGB289. Usher II is primarily caused by mutations in three genes with *USH2A* most commonly affected. This splicing allele is extremely rare as it has not been observed in population sequencing databases such as gnomAD. SpliceAI predicts the variant to create a novel splice donor site and a novel splice acceptor site upstream the predicted donor site, causing an in-frame insertion of a 129-bp-long cryptic exon. The cryptic exon contains a stop codon downstream of the cryptic acceptor splice site, resulting in the generation of a premature stop codon downstream of amino acid position 2,894 out of a total of 5,202 amino acids in the wild-type protein. There are three annotated functional domains downstream of position 2,894, including a fibronectin III domain, a transmembrane domain, and a PDZ1 domain. In addition, numerous pathogenic mutations have been reported downstream of exon 44 (McGee et al., 2010). Consistent with the prediction, *in vitro* minigene assay showed that constructs containing the c.8682-654C>G variant produced two splicing isoforms (Figure 2A). Based on the gel band intensity, we estimated the relative abundance of the two RT-PCR products at 72 and 28%, respectively. The major isoform contains the original exon and a cryptic exon of 129 bp, which exactly matched *in silico* prediction as confirmed by Sanger sequencing (Figure 3A). Consistent with the idea that this novel splicing mutation is likely to be the causal mutation in the proband, additional coding mutations have been identified in *USH2A* in both probands. Patient DGB289 carries a missense pathogenic mutation in the coding region of *USH2A*, c.10073C>T (p.C3358Y), that has been previously reported (Garcia-Garcia et al., 2011; Le Quesne Stabej et al., 2012; Zhao et al., 2015; Stone et al., 2017). Similarly, in patient MEP337, a previously reported nonsense coding mutation in *USH2A*, c.11864G>A (p.W3955X), has been identified (Lenarduzzi et al., 2015; Likar et al., 2018). The clinical phenotypes of both patients lend support to the pathogenicity of their *USH2A* mutations. Segregation analysis was performed for MEP337 (Figure 1C). The parents and brothers of MEP337 are asymptomatic. The mother of MEP337 is heterozygous of the coding variant, c.11864G>A, while the father carries neither allele identified in MEP337. Consequently, the splicing variant c.8682-654C>G is caused by *de novo* mutation. Patient MEP337, diagnosed with Usher II, demonstrated decreased night vision and congenital hearing loss of 30% in both ears (Figure 1C). Patient DGB289, who was diagnosed with RP, presented night vision difficulties and a decrease in the visual field (Figure 1D). Additionally, this patient has no family history of progressive retinal degeneration.

MEP344 is a 17-year-old male who was diagnosed with optic atrophy type 1 (Figure 1E) and has no family history. In this patient, a heterozygous variant (c.1608+622A>G) was found in intron 16 of *OPA1*, mutations in which lead to autosomal dominant optic atrophy (ADOA). This variant is extremely rare in the population as it has not been observed in genome sequencing databases such as gnomAD. The variant was predicted to result in the formation of a novel splicing donor site downstream of exon 16, causing an in-frame-insertion of a new cryptic exon of 54 bp between exons 16 and 17. Exon 16 and exon 17 reside in a GTPase domain (exons 9–16) and a linker region (exons 17–18), respectively, in which more than 25% of reported mutations are localized (Li et al., 2019). Although the insertion of the cryptic exon was not predicted to result in a shift in the open reading frame, an insertion of 18 amino acids right between the essential GTPase domain and the linker region is likely to affect the normal functionality of OPA1 protein. Minigene assay confirmed the SpliceAI prediction, as a transcript of the 54-bp-long cryptic exon was generated (Figures 2B, 3B). Indeed, many pathogenic mutations have been reported around exons 16 and 17 (Toomes et al., 2001; Le Roux et al., 2019). Taken together, this allele is likely to be pathogenic.

Patient DGB288 was diagnosed with Usher II (Figure 1F; Supplementary Figure S3). In patient DGB288, we identified two heterozygous variants in *ADGRV1* (c.5965dupA and c.14661+717A>G). The *ADGRV1* splicing variant c.14661+717A>G is a novel variant, and it has not been observed in genome sequencing databases such as gnomAD. It was predicted to contribute to the usage of cryptic donor and acceptor splice sites deep inside intron 71, leading to a non-frameshift insertion of an 84-bp-long cryptic exon downstream of exon 71. Interestingly, the minigene assay result of this splicing candidate did not fully agree with *in silico* predictions, as a 119-bp-long cryptic exon was included instead (Figures 2C, 3C). The relative abundance of the normal and aberrant transcripts generated by the minigene construct containing the variant was 37 vs. 63%, respectively. A premature stop codon is introduced 17 amino acids downstream of the cryptic exon’s acceptor site as a result of the mutation, presumably triggering nonsense-mediated decay (NMD) or deleting both the GPCR autoproteolysis inducing domain (GAIN) and the transmembrane domains (Sun et al., 2013). Moreover, many pathogenic mutations have been reported downstream of exon 71 (Richards et al., 2015; Sun et al., 2018). Taken together, the c.14661+717A>G variant is a likely pathogenic mutation leading to Usher II. The second mutation observed in the patient (c.5965dupA) is a pathogenic frameshift mutation in the coding region with a very low population frequency of 1.43 × 10.5. It causes an early frameshift (codon position 1,990 out of 6,306 in total), resulting in NMD or a severe truncation of ADGRV1 protein.

Patient NEI4320 was diagnosed with cone-rod dystrophy (Figure 1G). WGS identified two heterozygous variants in *RPGRIP1*, a coding variant c.2114A>C in exon 14 and a splicing variant c.2367+82A>G in intron 15. The nonsynonymous coding variant, c.2114A>C, is a novel variant that has not been reported in population databases like gnomAD, and multiple *in silico* algorithms predicted this variant to have a deleterious effect (CADD rank core = 0.78; VEST3 rank score = 0.91, GERP++ rank score = 0.74). This sequence change replaces a glutamine residue with a proline residue at codon 705 of the RPGRIP1 protein (p.Q705P), which is likely to impact secondary protein structure as there is a large physicochemical difference between the two residues. The intronic splicing variant c.2367+82A>G has not been observed in the genome sequencing database either, and it was predicted to activate a downstream cryptic donor site, leading to an in-frame insertion of 81 bp between exons 15 and 16 that generates a premature stop codon 19 amino acid downstream of the original donor splice site of exon 15. The creation of a premature stop codon results in either NMD of mRNA or the production of a truncated protein that potentially lacks the functionality of normal RPGRIP1 proteins. Previous studies identified many nonsense mutations downstream of exon 16 of *RPGRIP1*, including p.R814X (Weisschuh et al., 2018) and p.981X (Carss et al., 2017). Based on *in vitro* minigene assay, the c.2367+82A>G variant produced two bands (Figure 2D), with a relative band intensity of 65 and 35%, respectively. The major band caused by this variant was composed of the aberrant transcript of the exon 15 and addition of 81 bp downstream, which exactly matched *in silico* prediction (Figure 3D), while the minor band was identified as the wild-type. Taken together, the c.2367+82A>G variant is a likely pathogenic mutation leading to cone-rod dystrophy.

In patient MEP105, two heterozygous variants were identified (c.1163G>T and c.3998+3023T>G), in exon 12 and intron 30 of PCDH15, respectively. Neither allele has been observed in the genome sequencing databases. The nonsynonymous sequence change caused by the coding variant c.1163G>T replaces a highly conserved leucine residue with a proline at codon 429 of the PCDH15 protein (p.L429P). In addition, multiple *in silico* algorithms predict this variant to have a deleterious effect (GERP++ rank score = 0.69; CADD rank score = 0.74; VEST3 rank score = 0.72). The splicing variant c.3998+3023T>G was predicted to create a cryptic acceptor site at the mutation site and a donor site 29 bp downstream of the novel acceptor site. The insertion of a 29-bp-long cryptic exon downstream of exon 30 results in a shift in the open reading frame, leading to the generation of a stop codon 98 bp downstream of the canonical acceptor site of exon 31. The premature stop codon production presumably results in NMD or truncated PCDH15 proteins without the transmembrane and cytoplasmic domain. Minigene assay confirmed the SpliceAI prediction, as a transcript of the 29-bp-long cryptic exon was generated (Figures 2E, 3E). Mutations in *PCDH15* have been associated with Usher syndrome type I (Usher I) and non-syndromic hearing loss. Patient MEP105 was diagnosed with Goldman Farve disease and did not present any hearing problem but complained about some decrease in vision and visual fields (Figure 1H).

## Discussion

Deep-intronic splicing variants that alter splicing patterns may affect protein functions and have a remarkable contribution to diseases. To identify missing noncoding variants in unsolved IRD cases, we performed WGS in 571 probands and identified one known and five novel deep-intronic variants in nine probands, representing 1.1% of the cohort. Four novel deep-intronic variants (c.14661+717A>G variant in *ADGRV1*, c.8682-654C>G variant in *USH2A*, c.1608+622A>G variant in *OPA1*, and c.3998+3023T>G variant in *PCDH15*) activate cryptic donor and acceptor splice sites close to the mutation sites and thereby result in cryptic exon inclusion. Variant c.2367+82A>G in *RPGRIP1* creates a cryptic donor site and leads to exon elongation. All five novel splicing variants are deleterious for the following reasons: (1) they are rare in population; (2) they are predicted to lead to frameshift, premature stop, disruption of functional domains, and likely mRNA NMD; (3) the predictions are further validated using the minigene assay; and (4) clinical phenotype is consistent with the molecular diagnosis. It is worth noting that out of the six deep intronic variants that we identified, only one has been reported previously, suggesting that a significant portion of cryptic splicing mutations remain undiscovered.

Interestingly, the c.8682-654C>G variant in *USH2A* appears in two unrelated patients with different clinical features. This discrepancy could be explained by the severity of different coding variants carried by the two patients. MEP337, carrying a loss-of-function (LOF) frameshift coding mutation, was born with congenital sensorineural hearing loss and diagnosed with Usher II. DGB289, carrying a nonsynonymous mutation that is likely to be a hypomorph allele, exhibiting a milder phenotype with nonsyndromic RP.

Another interesting case is MEP105. The patient MEP105 was diagnosed with RP, and we identified two novel deleterious alleles (c.1163G>T and c.3998+3023T>G). The coding variant is a missense variant that is likely to cause a partial loss of function of PCDH15. The splicing variant is likely to be a severe allele, as it is likely to cause LOF frameshift, and complete splicing was observed based on minigene assay results. However, mutations in *PCDH15* have so far been associated with Usher I and nonsyndromic recessive hearing loss (DFNB23), but not yet with nonsyndromic vision loss. Interestingly, patient MEP105 is reported to have only retinal dysfunction and a late disease onset at age 42, a much milder disease progression compared to that of USH1F. We hypothesized that the milder phenotype is due to a partial loss of function mutations carried by the patient. However, further investigation is needed as we cannot rule out the possibility that the patient phenotype is due to mutations in other IRD associated genes.

With a cutoff of 0.5, high specificity is achieved with all of the splicing mutations predicted by SpliceAI confirmed by minigene assays. Furthermore, in most cases, the predicted splicing junctions were also confirmed by minigene assay results. However, we did observe one inconsistency between the minigene test and predictions. Comparing the *in vitro* and *in silico* results, we observed a shift in the splicing junction in the case of c.14661+717A>G in *ADGRV1*. This difference might be due to the inaccuracy of either the *in silico* predictions or the *in vitro* functional assay. Further investigation is needed to resolve the discrepancy.

The prediction scores assigned to splice site disruption seem to positively correlate with the impact of mutations on splicing. Based on the minigene assay results, variant c.1608+622A>G in *OPA1*, and variant c.3998+3,023 T>G in *PCDH15* only generated aberrant splicing, while variant c.14661+717A>G in *ADGRV1*, variant c.8682-654C>G in *USH2A*, and variant c.2367+82A>G in *RPGRIP1* produced both normal and aberrant transcript. The variants showing complete abnormal splicing had high prediction scores on both donor and acceptor sites (all of them are ≥0.5), while the ones that presented partial cryptic splicing were predicted to have lower scores for acceptor gain sites (variant c.14661+717A>G in *ADGRV1* and variant c.8682-654C>G in *USH2A*) or donor loss sites (variant c.2367+82A>G in *RPGRIP1*).

With a high cut-off score, a high specificity of cryptic splicing mutation can be achieved. However, it is likely that a significant proportion of deep intronic cryptic splicing mutations was missed with our current cut off. To increase sensitivity, one could lower the threshold. However, as the SpliceAI RNA-seq validation rate and sensitivity are proportional to the prediction score (i.e., 20% at 0.2; 80% at 0.8; Jaganathan et al., 2019), the frequency of false-positives can increase dramatically. One potential way to circumvent this issue is to complement *in silico* prediction with high-throughput functional splicing assays. In addition, although SpliceAI is quite accurate given the current threshold, improvement on splice site prediction is likely needed, especially for the variants with a lower score than 0.5. Our data and results of other studies alike can be used as positive control training sets for further improvements of predictions made by SpliceAI or other *in silico* tools. Another limitation of current splicing mutation prediction tools is that they do not predict the ratio of normal and aberrant splice isoforms, which is a critical factor to consider when assessing the strength of the mutant allele. Therefore, further improvement on *in silico* prediction and high-throughput functional assays might be needed if we would like to enable efficient large-scale examination of intronic variants’ impact on splicing.

In conclusion, the identification of five novel noncoding splicing variants highlights the relevance of discovering hidden deleterious variants in noncoding regions that alter splicing to increasing the genetic diagnostic yield of IRDs. Given that five out of six variants we identified were novel, the contribution of intronic variants, especially those deep within introns, to genetic diseases might be underestimated. Therefore, massively parallel approaches that can effectively characterize splicing-altering sequence variation have great potentials to accelerate the discovery process, facilitating clinical molecular diagnoses by identifying abundant pathogenic non-canonical splicing variants as the cause of human diseases.

## Data Availability Statement

The datasets for this article are not publicly available due to concerns regarding participant/patient anonymity. Requests to access the datasets should be directed to the corresponding author.

## Ethics Statement

The studies involving human participants were reviewed and approved by Baylor College of Medicine. Written informed consent to participate in this study was provided by the participants’ legal guardian/next of kin. Written informed consent was obtained from the individual(s), and minor(s)’ legal guardian/next of kin, for the publication of any potentially identifiable images or data included in this article.

## Author Contributions

XQ and RC designed the study. XQ was responsible for writing, collecting data, analysis, interpretation, and revision of the present article. JW and MW were responsible for data collecting and analysis partly. AI, KJ, DB, KG, and MP were responsible for clinical data collection and analysis. All authors contributed to the writing and revision of the manuscript. The final manuscript was approved by all authors.

### Conflict of Interest

The authors declare that the research was conducted in the absence of any commercial or financial relationships that could be construed as a potential conflict of interest.
