# Supplementary material for: Identification of Deep-Intronic Splice Mutations in a Large Cohort of Patients With Inherited Retinal Diseases
Source: Front Genet. 2021 Mar 2;12:647400. doi: 10.3389/fgene.2021.647400 (PMC7960924; doi:10.3389/fgene.2021.647400)
Supplement: Supplementary file 1 [file Data_Sheet_1.pdf]

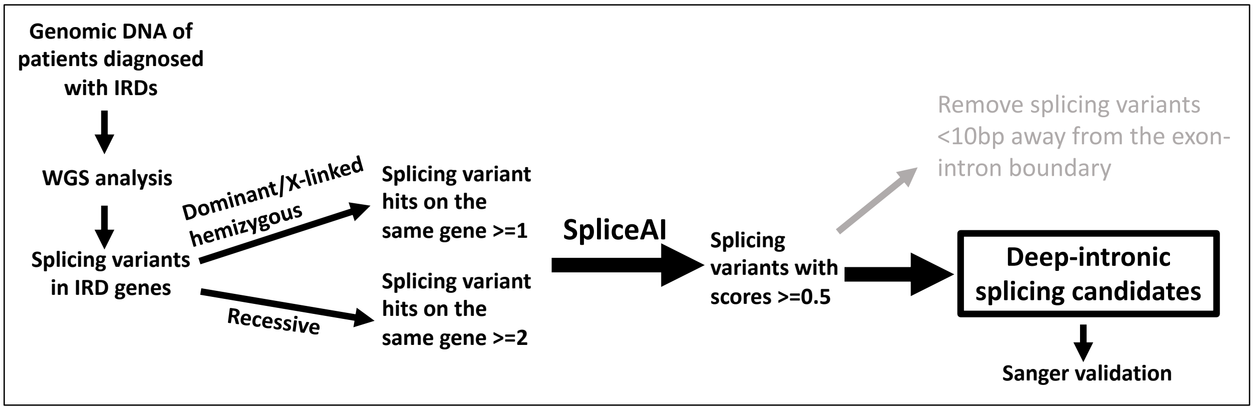

Supplementary Figure 1: Flowchart of prioritization scheme for candidate deep-intronic splicing variants

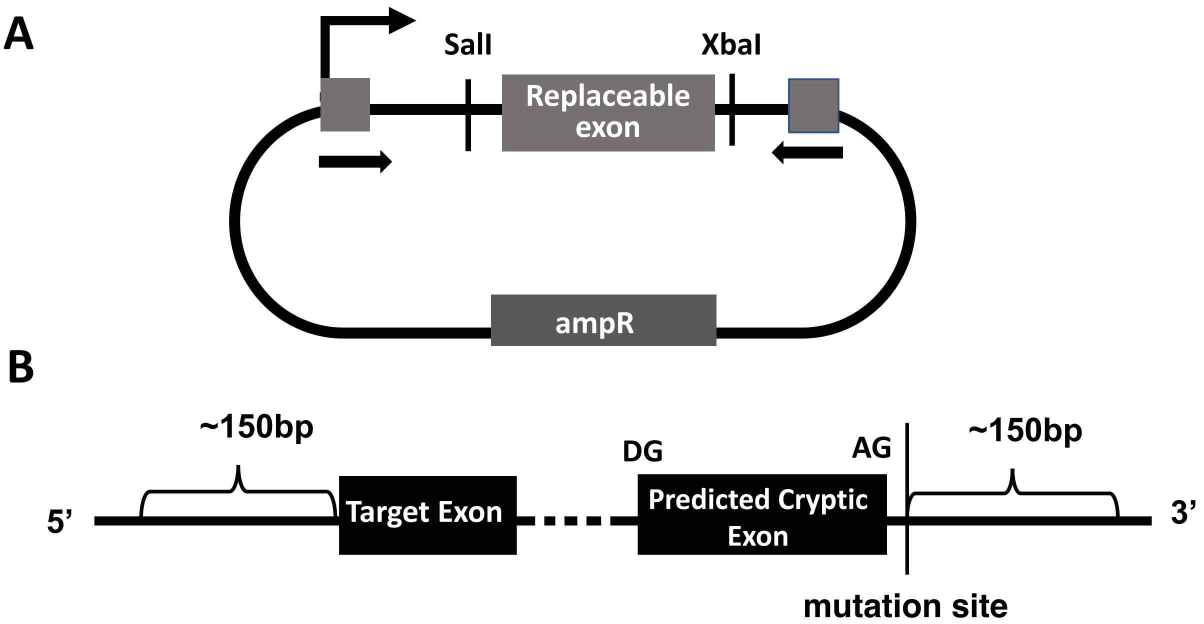

Supplementary Figure 2: a) Schematic of RHCglo minigene design and b) Schematic of genomic region of patient DNA that was PCR amplified and used to replace the replaceable exon between the SalI and XbaI restriction enzyme sites in the RHCglo vector. About 150 base pairs upstream and downstream of target exon/predicted cryptic exon/mutation site were included for PCR amplification. DG, predicted donor site position given by SpliceAI; AG, predicted acceptor site position predicted by SpliceAI

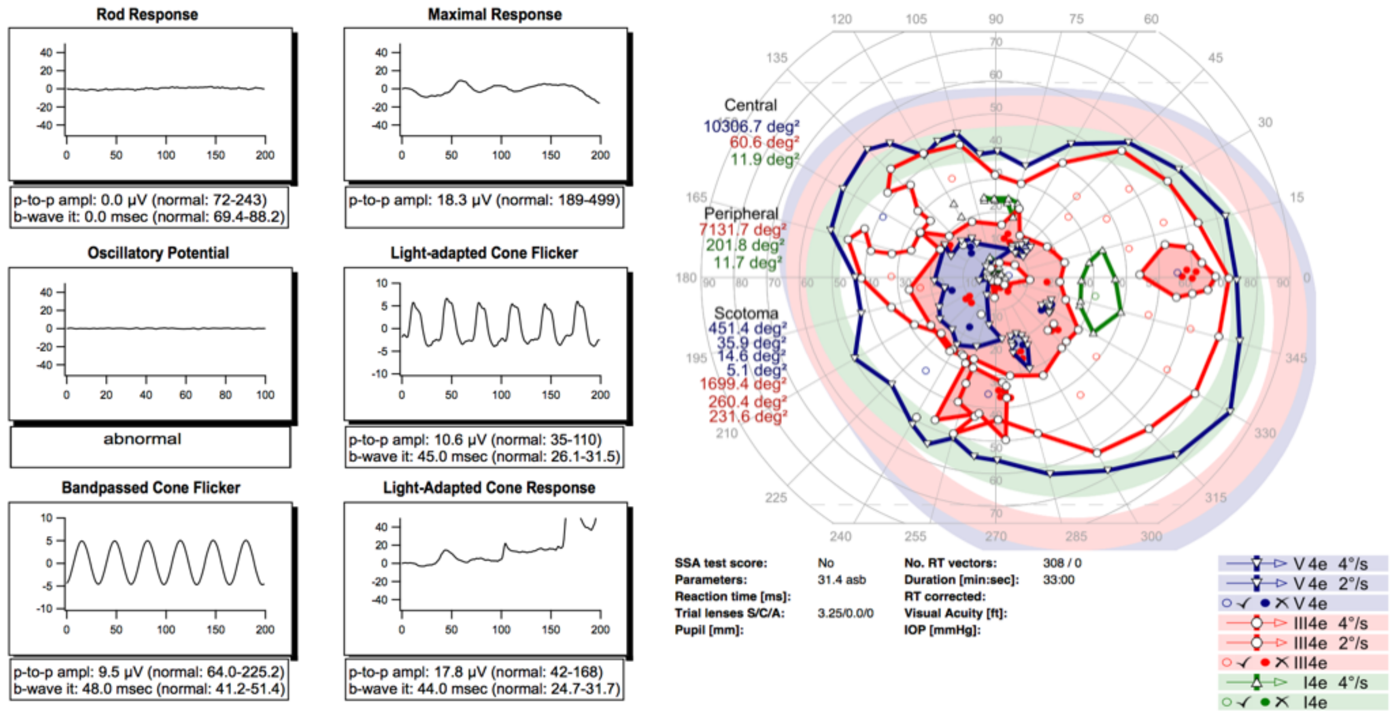

Supplementary Figure 3: Full-field ERGs (ffERGs) and kinetic perimetry of patient DGB288. Visual fields of the right eye showed a pericentral scotoma, constricting the central field to less than 10 degrees, and scotomas in the temporal and inferior periphery. ffERGs were obtained from the left eye. Rod responses to single flashes of white light were non-detectable. Cone responses to 30 Hz flicker were reduced in amplitude by 45% and significantly delayed in b-wave implicit time.

chromosomal position	DS_AG	DS_AL	DS_DG	DS_DL	DP_AG	DP_AL	DP_DG	DP_DL
chr1:216041166:C>G	0.36	0	0.5	0.01	131	279	3	-50
chr8:87617644:C>T	0.81	0	0.9	0	37	218	4	0
chr10:55597057:A>C	0.55	0	0.61	0	0	-28	-28	2
chrX:46736926:G>A	0.56	0.59	0.02	0.14	2	14	-409	99
chr14:21793624:A>G	0.02	0.01	0.69	0.22	-233	-230	-1	-82
chr5:90099416:A>G	0.19	0.01	0.79	0.01	-84	-376	-1	-71

Supplementary Table 1: SpliceAI prediction scores of identified 6 deep-intronic splicing variants. Delta score of a variant, defined as the maximum of (DS_AG, DS_AL, DS_DG, DS_DL), is interpreted as the probability of the variant being splice-altering. DS_AG: delta score of acceptor gain; DS_AL: delta score of acceptor loss; DS_DG: delta score of donor gain; DS_DL: delta score of donor loss; DP_AG: delta position of acceptor gain site relative to the mutation site; DP_AL: delta position of acceptor loss site relative to the mutation site; DP_DG: delta position of donor gain site relative to the mutation site; DP_DG: delta position of donor loss site relative to the mutation site.

Supplementary Table 2: Primer sequences

MEP105_F	GGGGTCGACgataatgccagtgcccaagt
MEP105_R	GTCTAGAgcagagtcagaacggaacaa
MEP337_F	GGGGTCGACtgatctgaggccagtgtttg
MEP337_R	GTCTAGAtgtacatgggggaggttcat
MEP344_F	GGGGTCGACttttgccttctcttcgtattca
MEP344_R	GTCTAGAgggatgagaatccctccttt
DGB288_F	GGGGTCGACtttgtgatggggaaaagtgc
DGB288_R	GTCTAGAccaaatgaatggttttgctg
DGB288_mut_F	ATATGGGCAGGTATAGTCTCTGG
DGB288_mut_R	TCCTGCCCATTCATCTGT
DGB289_F	GGGGTCGACtgatctgaggccagtgtttg
DGB289_R	GTCTAGAtgtacatgggggaggttcat
NEI4320_F	GGGGTCGACtcctaagcaccaatgcagaa
NEI4320_R	GTCTAGAatcttcacctgcctgcaaac

Note: the same set of primers were used for Sanger validation of variant authenticity and PCR amplification of patient DNA
